# Adropin Contributes to Anti-Atherosclerosis by Suppressing Monocyte-Endothelial Cell Adhesion and Smooth Muscle Cell Proliferation

**DOI:** 10.3390/ijms19051293

**Published:** 2018-04-26

**Authors:** Kengo Sato, Tomoyuki Yamashita, Remina Shirai, Koichiro Shibata, Taisuke Okano, Maho Yamaguchi, Yusaku Mori, Tsutomu Hirano, Takuya Watanabe

**Affiliations:** 1 Laboratory of Cardiovascular Medicine, Tokyo University of Pharmacy and Life Sciences, 1432-1 Horinouchi, Hachioji-City, Tokyo 192-0392, Japan; s126208@toyaku.ac.jp (T.Y.); s106101@toyaku.ac.jp (R.S.); s149064@toyaku.ac.jp (K.S.); s149021@toyaku.ac.jp (T.O.); s139110@toyaku.ac.jp (M.Y.); watanabe@toyaku.ac.jp (T.W.); 2 Division of Diabetes, Metabolism, and Endocrinology, Department of Medicine, Showa University School of Medicine, 1-5-8 Hatanodai, Shinagawa-ku, Tokyo 142-8666, Japan; torigoe1234@yahoo.co.jp (Y.M.); hirano@med.showa-u.ac.jp (T.H.)

**Keywords:** atherosclerosis, endothelial cell, inflammation, macrophage, monocyte, smooth muscle cell

## Abstract

Adropin, a peptide hormone expressed in liver and brain, is known to improve insulin resistance and endothelial dysfunction. Serum levels of adropin are negatively associated with the severity of coronary artery disease. However, it remains unknown whether adropin could modulate atherogenesis. We assessed the effects of adropin on inflammatory molecule expression and human THP1 monocyte adhesion in human umbilical vein endothelial cells (HUVECs), foam cell formation in THP1 monocyte-derived macrophages, and the migration and proliferation of human aortic smooth muscle cells (HASMCs) in vitro and atherogenesis in *Apoe*^−/−^ mice in vivo. Adropin was expressed in THP1 monocytes, their derived macrophages, HASMCs, and HUVECs. Adropin suppressed tumor necrosis factor α-induced THP1 monocyte adhesion to HUVECs, which was associated with vascular cell adhesion molecule 1 and intercellular adhesion molecule 1 downregulation in HUVECs. Adropin shifted the phenotype to anti-inflammatory M2 rather than pro-inflammatory M1 via peroxisome proliferator-activated receptor γ upregulation during monocyte differentiation into macrophages. Adropin had no significant effects on oxidized low-density lipoprotein-induced foam cell formation in macrophages. In HASMCs, adropin suppressed the migration and proliferation without inducing apoptosis via ERK1/2 and Bax downregulation and phosphoinositide 3-kinase/Akt/Bcl2 upregulation. Chronic administration of adropin to *Apoe*^−/−^ mice attenuated the development of atherosclerotic lesions in the aorta, with reduced the intra-plaque monocyte/macrophage infiltration and smooth muscle cell content. Thus, adropin could serve as a novel therapeutic target in atherosclerosis and related diseases.

## 1. Introduction

Atherosclerosis is regarded as a chronic inflammatory disease in response to injury of the arterial wall in which the inside stenosis is due to plaque formation [1]. Vascular inflammation stimulates the expression of adhesion molecules, such as intercellular adhesion molecule 1 (ICAM1), vascular cell adhesion molecule 1 (VCAM1), and selectin E, in endothelial cells (ECs). These effectors encourage monocyte adhesion to ECs and monocyte infiltration into the subendothelial space, followed by fatty streak formation with the accumulation of lipid-laden macrophage foam cells [1,2]. Foam cell formation is characterized by the intracytoplasmic accumulation of cholesterol ester, which depends on the balance between the uptake of oxidized low-density lipoprotein (LDL) via scavenger receptor class A (SRA) and the efflux of free cholesterol controlled by the ATP-binding cassette transporter A1 (ABCA1) [2]. The accumulation of cholesterol ester is also regulated by the balance between cholesterol esterification by acyl-coenzyme A: cholesterol acyltransferase 1 (ACAT1) and its hydrolysis by neutral cholesterol ester hydrolase (NCEH) [2]. Macrophage phenotypes classified as pro-inflammatory M1 and anti-inflammatory M2 have recently focused on atherosclerosis [3]. In addition, the migration and proliferation of vascular smooth muscle cells (VSMCs) and their production of extracellular matrix (ECM) components, such as the collagen 1, collagen 3, fibronectin, elastin, and matrix metalloproteinases (MMPs), all contribute to the development of the atherosclerotic plaque [1].

**Figure 1:**
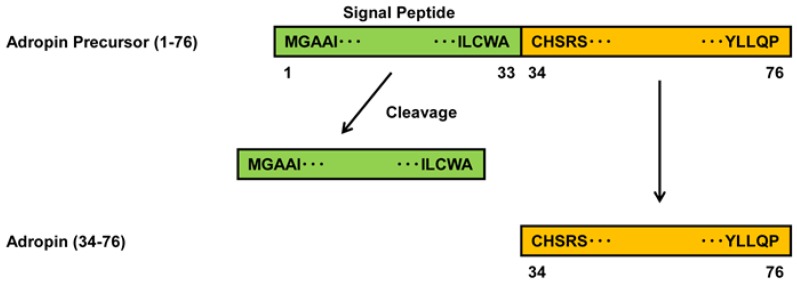
Biosynthesis of adropin. Adropin precursor protein consisting of 76 amino acids produces the 43 amino acid adropin by the cleavage of a 33 amino acid signal peptide.

Adropin was identified in liver and brain as a new metabolic hormone that serves to modulate lipid and glucose metabolism [4]. Adropin is a secreted protein encoded by the energy homeostasis associated (*ENHO*) gene [4]. Adropin precursor protein contains 76 amino acids that include the 43 amino acid peptide hormone and a 33 amino acid signal peptide (Figure 1) [4]. Human, mouse, and rat adropin amino acid sequences are 100% identical [4]. The specific receptor for adropin has not yet been identified but G protein-coupled receptor 19 (GPR19) is regarded as a putative receptor [5]. Adropin is expressed in liver, brain, heart, kidney, pancreas, human umbilical vein ECs (HUVECs), and coronary artery ECs [4,6,7]. Adropin inhibits tumor necrosis factor α (TNFα)-induced apoptosis and promotes migration, proliferation, and permeability in HUVECs via increasing the expression of endothelial nitric oxide synthase (NOS) [6]. Adropin reduces the permeability in rat brain microvascular ECs under ischemic conditions (hypoxia/low glucose) [8]. Adropin also preserves the blood–brain barrier function for intracerebral hemorrhage via the Notch1 signaling pathway in mice [9]. Adropin ameliorates murine limb perfusion and increases capillary density following hindlimb ischemia in mice [6]. Several lines of clinical evidence have shown that blood adropin levels are decreased in coronary artery disease (CAD), diabetes, metabolic syndrome, and obesity, and correlated negatively with the severity of CAD, body mass index, age, homeostasis model assessment of insulin resistance (HOMA-IR), insulin and homocysteine levels, and endothelial dysfunction in patients with diabetes, metabolic syndrome, and obesity [10,11,12,13,14,15,16,17,18,19,20,21]. However, the effects of adropin on atherogenesis have not yet been reported.

In the present study, we aimed to clarify the effects of adropin, in vitro, on TNFα-induced adhesion of human THP1 monocytes to HUVECs and angiotensin II (AngII)-induced migration, proliferation, and ECM production in human aortic smooth muscle cells (HASMCs). We also investigated the molecular mechanisms by assessing the adhesion molecules in HUVECs, the inflammatory phenotype in THP1 monocyte-derived macrophages, and the intracellular signal transduction in HASMCs, as well as macrophage foam cell formation. The in vivo studies focused on the development of aortic atherosclerotic lesions in *Apoe*^−/−^ mice.

## 2. Results

### 2.1. Expression of Adropin in Human Vascular Cells

**Figure 2:**
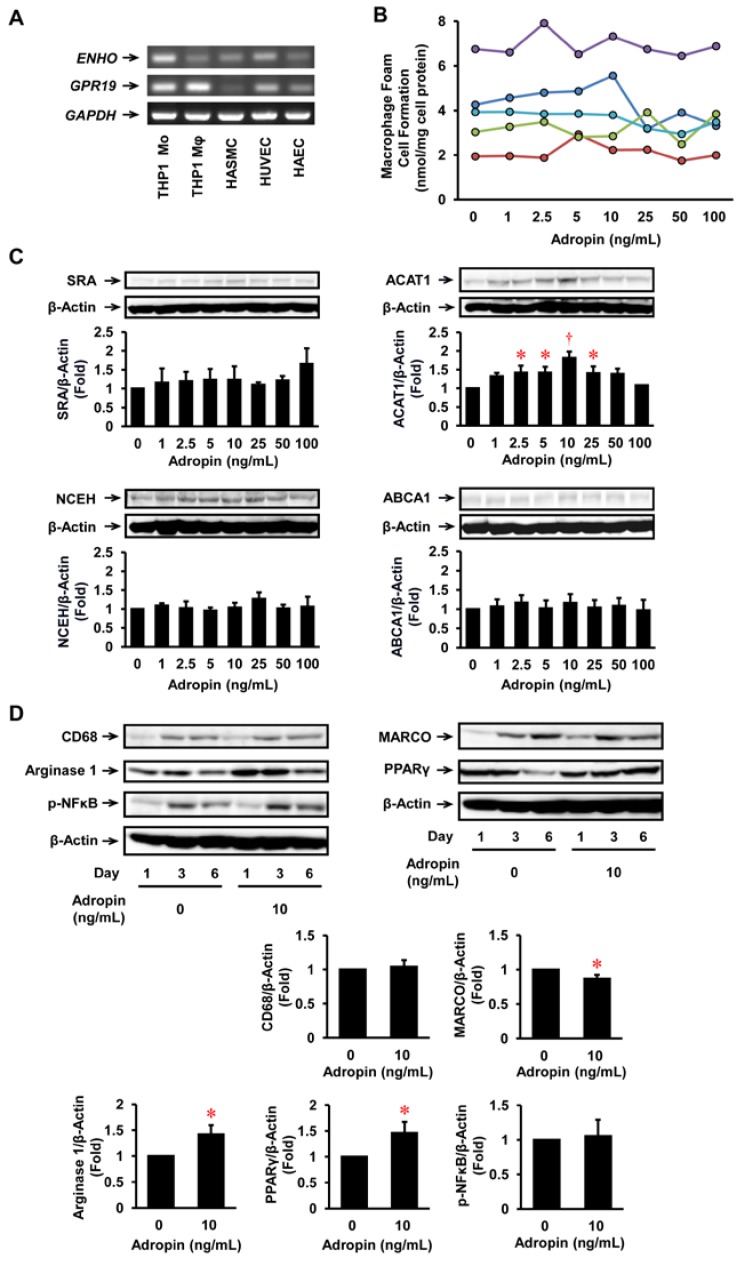
Expression of *ENHO* (adropin gene) and *GPR19* in human vascular cells and the effect of adropin on foam cell formation and inflammatory phenotype in THP1 monocyte-derived macrophages. (**A**) mRNA expression levels of *ENHO* and *GPR19* in THP1 monocytes, their derived macrophages, HASMCs, HUVECs, and HAECs were analyzed by RT-PCR. Glyceraldehyde-3-dehydrogenase (*GAPDH*) served as a loading control. Independent experiments were repeated twice to assure reproducibility. (**B**) THP1 monocytes were incubated for 6 days with the indicated concentrations of adropin, followed by a 19-h incubation with 50 μg/mL oxidized LDL in the presence of 100 μmol/L [^3^H]oleate. Foam cell formation was determined from reads of the intracellular radioactivity of cholesterol-[^3^H]oleate (*n* = 5). The results from 5 independent experiments are shown in different colors. (**C**) THP1 monocyte-derived macrophages cultured for 6 days were harvested before the addition of oxidized LDL for immunoblot for SRA, ACAT1, NCEH, ABCA1, and β-actin (*n* = 5–6). (**D**) THP1 monocytes were incubated for the indicated times with or without adropin (10 ng/mL). Cells were subjected to immunoblot for CD68 (a macrophage differentiation marker), MARCO (an M1 macrophage marker), arginase 1 (an M2 macrophage marker), p-NFκB, PPARγ, or β-actin (*n* = 3–4). The graph shows the expressions on day 6. * *p* < 0.05, ^†^ *p* < 0.005 vs. 0 ng/mL of adropin.

*ENHO* (adropin gene) was expressed at high levels in THP1 monocytes and HUVECs, and at low levels in THP1 monocyte-derived macrophages, HASMCs, and human aortic ECs (HAECs) (Figure 2A). *GPR19* was expressed at high levels in THP1 monocytes, macrophages, HUVECs, and HAECs, but at insubstantial levels in HASMCs (Figure 2A).

### 2.2. Effects of Adropin on Foam Cell Formation in Human Monocyte-Derived Macrophages

Adropin had no significant effects on oxidized LDL-induced foam cell formation and protein expression of SRA, NCEH, and ABCA1 in THP1 monocyte-derived macrophages (Figure 2B,C). However, adropin significantly increased ACAT1 protein expression (Figure 2C).

### 2.3. Effects of Adropin on Inflammatory Phenotypes in Human Monocyte-Derived Macrophages

Within 6 days after the start of THP1 monocyte culture, the differentiation of monocytes to macrophages was confirmed by increased protein expression of CD68, a macrophage differentiation marker (Figure 2D). Adropin did not affect significantly the differentiation from monocytes to macrophages. However, adropin significantly suppressed the protein expression of MARCO, an M1 marker, and enhanced that of arginase 1, an M2 marker, on day 6 (Figure 2D). Adropin also significantly increased peroxisome proliferator-activated receptor γ (PPARγ) expression and showed no significant effects on nuclear factor κB (NFκB) expression on day 6 (Figure 2D). These observations indicate that adropin shifted the macrophage phenotype overwhelmingly to the M2 rather than M1 phenotype associated with PPARγ upregulation during monocyte differentiation into macrophages.

### 2.4. Effects of Adropin on the Inflammatory Response in Human ECs

**Figure 3:**
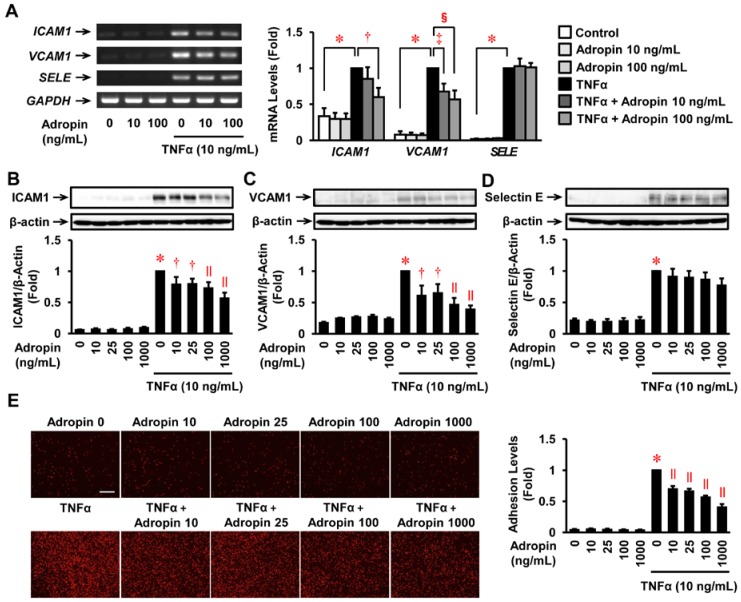
Effects of adropin on inflammatory response and monocyte adhesion in HUVECs. (**A**) mRNA expression of *ICAM1*, *VCAM1*, and *SELE* (selectin E gene) was analyzed by RT-PCR. HUVECs were pre-treated with adropin (0, 10, 100 ng/mL) for 30 min and then incubated with adropin (0, 10, 100 ng/mL) + TNFα (0, 10 ng/mL) for 4 h. Representative images are shown; the graph on the right side indicates densitometry data following normalization relative to *GAPDH* (*n* = 3–4). (**B**–**D**) HUVECs treated as described above were harvested and subjected to immunoblot to evaluate ICAM1, VCAM1, and selectin E protein expression (*n* = 4–5). Upper panels show representative immunoblots with densitometry data after normalization relative to β-actin shown beneath. (**E**) Confluent HUVECs were incubated in 0.5% fetal bovine serum (FBS)-EGM-2 for 16 h, and then pre-treated for 30 min with the indicated concentrations of adropin, followed by a 4-h incubation in the presence or absence of TNFα (10 ng/mL). Subsequently, calcein red-orange-labeled THP1 monocytes were plated on the HUVEC monolayer and incubated for 1 h. After washing, the adherent cells were observed by fluorescence microscopy (*n* = 4). Scale bar = 100 μm. Baseline (1 fold) = 66318.5 ± 4599.2 pixels. (**B**–**E**) * *p* < 0.0001 vs. corresponding control of TNFα (−); ^†^ *p* < 0.05, ^‡^ *p* < 0.001, ^§^ *p* < 0.005, ^||^ *p* < 0.0001 vs. corresponding control of TNFα (+).

Adropin had no significant effects on mRNA expression of *ICAM1*, *VCAM1*, and *SELE* (selectin E gene). Treatment with TNFα (10 ng/mL) significantly enhanced their expression levels (Figure 3A). However, adropin concentration-dependently suppressed TNFα-induced mRNA expression of *ICAM1* and *VCAM1*, but not *SELE*, with a significant inhibition observed at 100 ng/mL (Figure 3A). Immunoblots also revealed that TNFα (10 ng/mL) significantly increased protein expression of ICAM1, VCAM1, and selectin E, but adropin significantly in a concentration-dependent manner suppressed TNFα-stimulated upregulation of ICAM1 and VCAM1, but not selection E, in HUVECs (Figure 3B–D). The changes identified at the protein level were consistent with alterations in gene expression (Figure 3A).

### 2.5. Effects of Adropin on Human Monocyte Adhesion to Human ECs

Cytotoxic effects of adropin on THP1 monocytes and HUVECs were not observed at any concentration up to 1000 ng/mL. Adropin had no significant effect on THP1 monocyte adhesion to HUVECs (Figure 3E). Exposure of HUVECs to TNFα (10 ng/mL) for 4 h resulted in a 26-fold increase in THP1 monocyte adhesion compared with the untreated control (Figure 3E). However, this effect was significantly reduced by adropin at 10–1000 ng/mL, with a maximum reduction of 59% at 1000 ng/mL (Figure 3E).

### 2.6. Effects of Adropin on Migration, Proliferation, Apoptosis, ECM Expression, and Relevant Signal Transduction in HASMCs

**Figure 4:**
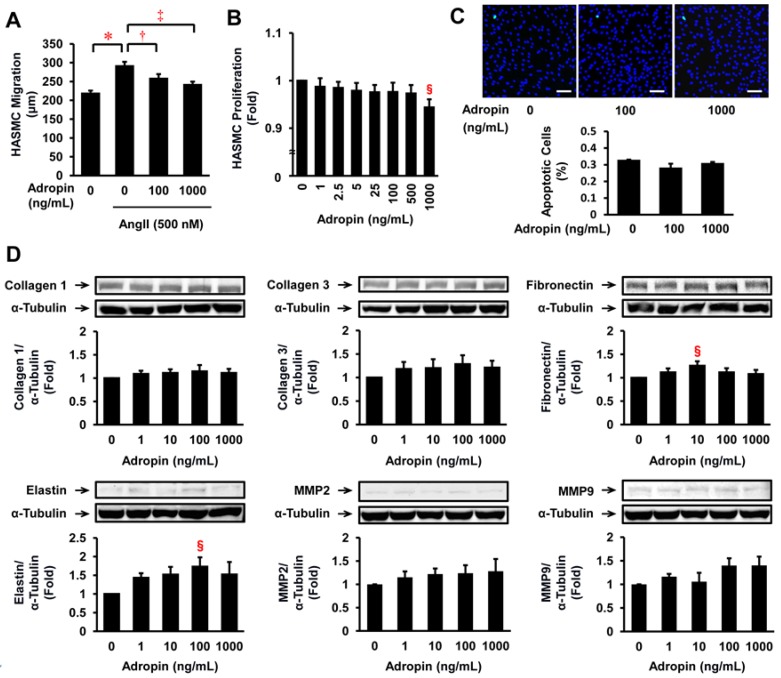
Effects of adropin on migration, proliferation, apoptosis, and ECM expression in HASMCs. (**A**) Migration was determined in 10 HASMCs per plate using a BIOREVO BZ-9000 microscope in serum-free SmGM-2 with or without AngII (500 nmol/L) and adropin (0, 100, 1000 ng/mL). Four independent experimental replicates were performed. * *p* < 0.0001 vs. 0 ng/mL of adropin. ^†^ *p* < 0.0005, ^‡^ *p* < 0.0001 vs. AngII. (**B**) The proliferation was determined by WST-8 assay following a 48-h incubation of HASMCs in 5% FBS-SmGM-2 with the indicated concentrations of adropin (*n* = 5). **^§^** *p* < 0.05 vs. 0 ng/mL of adropin. (**C**) HASMCs were stained as apoptotic cells (green) using the TUNEL method after a 48-h incubation in 5% FBS-SmGM-2 with the indicated concentrations of adropin. Nuclei were co-stained with 6-diamidino-2-phenylindole (blue). The graph indicates the percentage of apoptotic cells (*n* = 3). Scale bar = 100 μm. (**D**) HASMCs were incubated for 24 h in serum-free SmGM-2 with the indicated concentrations of adropin, and then harvested for immunoblot of collagen 1, collagen 3, fibronectin, elastin, MMP2, MMP9, and α-tubulin. Representative data showing protein expression (upper panels) with densitometry following normalization relative to α-tubulin (lower panels, *n* = 5–6). **^§^** *p* < 0.05 vs. 0 ng/mL of adropin.

Treatment with AngII (500 nmol/L) significantly increased the migration of HASMCs (Figure 4A). However, this effect was significantly suppressed by adropin at 100 and 1000 ng/mL (Figure 4A). Adropin at 1000 ng/mL significantly suppressed the proliferation of HASMCs (Figure 4B); apoptosis determined by terminal deoxynucleotidyl transferase-mediated deoxyuridine triphosphate-biotin nick end labeling (TUNEL) staining was not evident at this concentration (Figure 4C). Adropin significantly increased protein expression of fibronectin and elastin but not collagen 1, collagen 3, MMP2, or MMP9 in HASMCs (Figure 4D).

**Figure 5:**
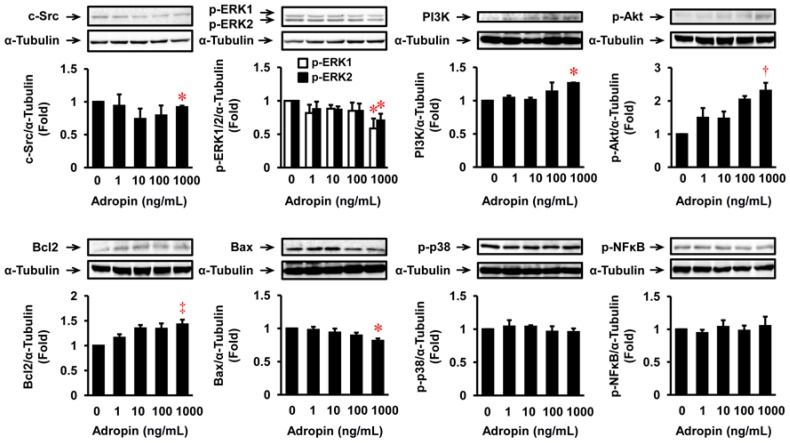
Effects of adropin on intracellular signal transduction in HASMCs. HASMCs were incubated for 24 h in 5% FBS-SmGM-2 with the indicated concentrations of adropin. The effects of adropin on intracellular signals were assessed by immunoblots. Representative data showing protein expression or phosphorylation (upper panels) with densitometry following normalization relative to α-tubulin (lower panels, *n* = 3–4). * *p* < 0.05, ^†^ *p* < 0.001, ^‡^ *p* < 0.005 vs. 0 ng/mL of adropin.

Next, the intracellular signal transduction pathways regarding the effects of adropin on VSMC responses were investigated. Treatment with adropin at 1000 ng/mL for 24 h significantly suppressed c-Src and Bax expression and ERK1/2 phosphorylation, and increased Bcl2 and phosphoinositide 3-kinase (PI3K) expression and Akt phosphorylation, without significant changes in p38 and NFκB phosphorylation in HASMCs (Figure 5).

### 2.7. Effects of Adropin on Atherosclerotic Lesion Development in Apoe^−/−^ Mice

**Figure 6:**
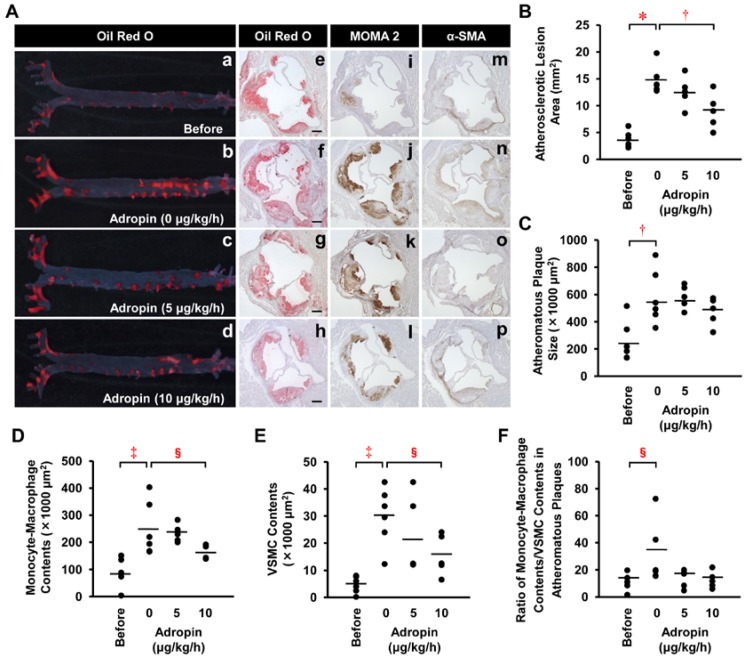
Effects of adropin on atherosclerotic lesion development in *Apoe*^−/−^ mice. Of the 22 *Apoe*^−/−^ mice at 17 weeks of age, six were sacrificed before infusion and six, five, and five were infused with adropin at doses of 0, 5, and 10 μg/kg/h using osmotic minipumps for 4 weeks. (**A**) The atherosclerotic lesions on the aortic internal surface were stained with Oil Red O (a–d). Cross-sections of the aortic sinus were stained with Oil Red O (e–h), with immunostains for monocyte-macrophage 2 (MOMA2; (i–l)) or α-smooth muscle actin (α-SMA; (m–p)). Scale bar = 200 μm. (**B**–**F**) Statistical comparisons of atherosclerotic lesion area, atheromatous plaque size, monocyte-macrophage or VSMC contents, and the ratio of monocyte-macrophage contents/VSMC contents within atheromatous plaques among four groups. * *p* < 0.0001, ^†^ *p* < 0.005, ^‡^ *p* < 0.0005, ^§^ *p* < 0.05. Bars indicate the mean values in the graphs.

In *Apoe*^−/−^ mice at 21 weeks old compared with 17 weeks old, the atherosclerotic lesion area of the aortic internal surface and the atheromatous plaque size of the aortic sinus wall, with intra-plaque monocyte-macrophage and VSMC contents, were significantly increased by 4.0, 2.3, 2.8, and 6.3-fold, respectively (Figure 6A(a,b,e,f,i,j,m,n),B–E). However, chronic infusion of adropin at 5 μg/kg/h did not significantly retard the increases in aortic atherosclerotic lesion area and atheromatous plaque size (Figure 6A(b,c,f,g),B,C). Notably, chronic infusion of adropin at 10 μg/kg/h significantly reduced the aortic atherosclerotic lesion area (Figure 6A(b,d),B), with a tendency to decrease the plaque size (Figure 6A(f,h),C), and significantly decreased the intra-plaque monocyte-macrophage and VSMC contents (Figure 6A(j,l,n,p),D,E). In addition, the ratio of monocyte-macrophage contents/VSMC contents within the atheromatous plaques, a biomarker of plaque instability, was markedly but not significantly decreased by adropin (Figure 6F).

There were no statistically significant differences in food intake, body weight, systolic and diastolic blood pressures, and plasma levels of total cholesterol, triglyceride, free fatty acid, glucose, and insulin, and HOMA-IR among the three groups of 21-week-old *Apoe*^−/−^ mice (Table 1).

## 3. Discussion

We provide the first evidence to show that adropin suppresses atherosclerosis. Adropin attenuates the inflammatory responses of ECs and monocyte-derived macrophages and the migration and proliferation of VSMCs. In addition to anti-atherosclerotic effects, adropin also increases protein expression of fibronectin and elastin in VSMCs. Fibronectin is a trigger for the recruitment of VSMCs in the formation of fibrous cap in atheromatous plaques [22]. Increased expression of fibronectin and elastin with adropin may contribute to adropin’s effect on plaque stability and vascular elasticity. The reason why adropin exerts multiple effects in all three vascular cells that participate in the pathogenesis of atherosclerosis could be explained by the possible presence of adropin’s own receptors, other than GPR19, in these cells. Each change in a variety of cellular and molecular phenomena induced by adropin in vitro seems to be minor. However, when all comes together, adropin exerts visibly anti-atherogenic effects in vivo. Ultimately, adropin infusion significantly retards the development of atherosclerotic lesions with reduced intra-plaque monocyte-macrophage and VSMC contents in the aorta of *Apoe*^−/−^ mice.

Molecular mechanisms for the effects of adropin on vascular cells have been rarely reported. During monocyte differentiation into macrophage, adropin decreases M1 phenotype acquisition and increases the expression of the M2 phenotype associated with PPARγ upregulation [3]. Adropin has no significant effect on macrophage foam cell formation despite ACAT1 upregulation. The action of ACAT1 may be opposed by a slight increase in NCEH expression in macrophages. In general, the migration and proliferation of VSMCs are regulated by the c-Src/ERK1/2 pathway [23,24]. The expression of fibronectin is mediated by the Akt pathway in VSMCs [25]. The present study suggests that adropin suppresses VSMC proliferation via the downregulation of the c-Src/ERK1/2 pathway and increases fibronectin and elastin expression probably via the upregulation of the PI3K/Akt pathway. It is possible that adropin may not induce apoptosis via both the upregulation of Bcl2, an anti-apoptotic molecule, and the downregulation of Bax, a pro-apoptotic molecule, in VSMCs.

In the present study, adropin is expressed in human ECs, THP1 monocytes, and their derived macrophages, and HASMCs. Adropin is also expressed abundantly in liver, pancreas, and fat and is related to adiposity and insulin resistance [4,7]. Knockout of adropin facilitates insulin resistance and worsens lipid metabolism in mice [26,27]. By contrast, overexpression and administration of adropin ameliorate glucose intolerance and improve lipid profiles in mice [4,28,29,30]. Adropin decreases mRNA expression of pro-inflammatory cytokines, such as TNFα and interleukin 6, via inducible NOS expression in pancreas and liver tissues [28,29]. Gu et al. reported that plasma adropin levels were significantly lower in hypertensive patients compared with normotensive controls [31]. Adropin had a negative correlation with diastolic and systolic blood pressures and plasma endothelin 1 levels [31]. Whereas Çelik et al. and Gulen et al. reported that hypertensive patients had higher adropin levels compared with controls [32,33]. Chen M. et al. reported that adropin is a novel regulator of blood pressure [34]. Previous studies have shown that adropin markedly upregulates phosphorylation of endothelial NOS and Akt through vascular endothelial growth factor receptor 2 in HUVECs [6]. However, adropin at the doses used in the present study showed no significant effects on blood pressure, plasma glucose level, lipid profile, and insulin resistance in *Apoe*^−/−^ mice. The present study indicates that adropin may prevent atherogenesis independently of glucose and lipid metabolism and blood pressure.

Recently, attention has been focused on non-lipid CAD biology [35]. The present study supports the evidence that adropin suppresses atherosclerosis by a non-lipid-driven mechanism. Adropin counteracts early vascular inflammation responses to injury including monocyte–endothelium interaction by inhibiting non-lipid inflammatory pathways. A genetic variant in *ENHO* is associated with rheumatic arthritis, another inflammatory phenotype [36], but this locus has not been found to associate with a metabolism phenotype. Therefore, the present study indicates the significance of adropin’s non-lipid role in CAD protection, which may represent a novel effect of adropin distinct from previous reports. In addition, clinical studies have recently shown that aerobic exercise and dietary fat intake increase the circulating blood levels of adropin in obese subjects [18,37,38], contributing to the prevention of CAD.

The physiologic relevance of the adropin concentrations used in our experiments warrants further discussion. First, the concentrations of adropin needed to influence the multiple responses in THP1 monocyte-derived macrophages, HUVECs, and HASMCs were considerably high (2-fold, at maximum ~196-fold) compared with average plasma concentration of adropin (5.12 ng/mL) in healthy volunteers [10]. According to our previous studies [39,40,41], atheroprotective agents are increased to counteract the development of atherosclerosis. The local levels of vasoactive agents could increase to a similar degree by the generation from vascular cells in an autocrine/paracrine fashion [42,43]. Next, the concentrations of adropin differed in terms of the influence on THP1 monocyte-HUVEC adhesion and relevant adhesion molecule expression in HUVECs, and HASMC migration and proliferation, and ECM production. This likely reflects the different cell types used, their intracellular signaling pathways, and the expression levels of GPR19. Because the expression level of GPR19 is very low in VSMCs, the cells may be ineffective unless its concentration goes up to a high level. Finally, several studies have shown that serum adropin concentrations are low in patients with CAD, metabolic syndrome, and type 2 diabetes [10,11,12,13,14,15]. In our present results, adropin is abundantly expressed in ECs, as well as monocytes, macrophages, and VSMCs. The reason may be attributed to the decrease of adropin production caused by vascular endothelial dysfunction due to the above diseases.

In conclusion, the results of the present study indicate that adropin exerts anti-atherosclerotic effects by suppressing the inflammatory responses in ECs and monocytes/macrophages, monocyte-EC adhesion, and the migration and proliferation of VSMCs. The results provide insight into the potential use of adropin to expand a therapeutic window in the prevention of atherosclerosis. Thus, the development of adropin analogs and receptor agonists may serve as potential therapeutic targets in atherosclerosis and its related diseases.

## 4. Materials and Methods

### 4.1. Foam Cell Formation Assay

THP1 monocytes (Health Science Research Resources Bank, Osaka, Japan) were seeded in 3.5-cm dishes (1 × 10^6^ cells/1 mL/dish). Cells were incubated at 37 °C in 5% CO_2_ humidified incubator for 3 days in RPMI-1640 medium containing 10% FBS, 0.05 mg/mL streptomycin, 50 U/mL penicillin, and the indicated concentrations of adropin (Peptide Institute, Osaka, Japan) in the presence of phorbol-12-myristate 13-acetate (150 ng/mL) to induce differentiation into macrophages. Subsequently, THP1 monocyte-derived macrophages were incubated for 3 days in the absence of phorbol-12-myristate 13-acetate for immunoblots [44,45]. After then, THP1-derived macrophages were further incubated for 2 days in the renewal medium with the same concentrations of adropin along with 50 μg/mL oxidized LDL and 100 μmol/L [^3^H]oleate (PerkinElmer, Yokohama, Japan) conjugated with bovine serum albumin [39,40,41,44,45,46,47,48,49]. Cellular lipids were extracted and the radioactivity of cholesterol-[^3^H]oleate was determined by thin-layer chromatography [50].

### 4.2. Reverse Transcription Polymerase Chain Reaction (RT-PCR)

HUVECs (Lonza, Walkersville, MD, USA) were incubated at 37 °C in 5% CO_2_ for 30 min with the indicated concentrations of adropin in EGM-2 (Lonza). Adropin and 10 ng/mL TNFα (PeproTech, Rocky Hill, NJ, USA) were then added to the media for a further 4 h [41]. Total RNA was extracted using a High Pure RNA Isolation Kit (Roche Diagnostics, Mannheim, Germany). Complementary DNAs were synthesized from isolated RNA templates using a High Capacity cDNA Reverse Transcription Kit (Applied Biosystems, Foster City, CA, USA). The mRNAs for *ICAM1*, *VCAM1*, *SELE*, and *GAPDH* were detected as described previously [40,41,44,45,46,47,48]. In addition, mRNA expression of *ENHO* and *GPR19* was evaluated in THP1 monocytes, their derived macrophages, HASMCs, HUVECs, and HAECs. The PCR products were separated by 2% agarose gel electrophoresis, and the band intensity was quantified by densitometry [40,41,44,45,46,47,48].

### 4.3. Monocyte Adhesion Assay

Confluent HUVECs in 24-well plates were incubated at 37 °C in 5% CO_2_ for 16 h with EGM-2, and then pre-treated for 30 min with the indicated concentrations of adropin, followed by a 4-h incubation with or without 10 ng/mL TNFα. THP1 monocytes (1 × 10^5^ cells) labeled with Cell trace™ calcein red-orange (Life Technologies, Carlsbad, CA, USA) were added to each well of HUVEC-seeded 24-well plates. After 1 h of incubation, cells were washed four times. THP1 monocytes bound to HUVECs were examined by fluorescence microscopy (IX70; Olympus, Tokyo, Japan). Their adhesion was analyzed using image analysis software (ImageJ; NIH, Bethesda, MD, USA) [41,45,46].

### 4.4. Migration Assay

HASMCs (Lonza) at passage 6–8 were seeded into 8-well culture slides (3 × 10^3^ cells/250 μL/well). Cells were incubated at 37 °C in 5% CO_2_ for 3–5 h in SmGM-2 (Lonza). Subsequently, HASMCs were incubated with the indicated concentrations of adropin in serum-free SmGM-2 for 30 min, followed by a 16-h incubation with the indicated concentration of AngII (Sigma, St. Louis, MO, USA) and/or the same concentrations of adropin. Cells were photographed at 10-min intervals. The migration distance was measured in 10 cells randomly chosen in each well using a time-lapse cell culture observation system (BIOREVO BZ-9000 microscope; Keyence, Osaka, Japan) [39,40,41,44,45,46,47,48,49].

### 4.5. Proliferation (Viability) Assay

HASMCs at passage 6–8 were seeded into 96-well plates (1 × 10^4^ cells/100 μL/well) and incubated for 24 h in SmGM-2. Cells were then incubated for a further 48 h in fresh media with the indicated concentrations of adropin. Ten microliters of WST-8 solution (Cell Count Reagent SF; Nacalai Tesque, Kyoto, Japan) were then added to each well [39,40,41,44,45,46,47,48,49]. After 1 h of incubation, the amount of formazan product was determined spectrophotometrically (450 nm) using a Sunrise Remote R™-micro plate reader (Tecan, Kawasaki, Japan) [39,40,41,44,45,46,47,48,49].

### 4.6. Apoptosis Assay

HASMCs were seeded into 12-well plates (3 × 10^5^ cells/1 mL/well) and incubated at 37 °C in a 5% CO_2_ gassed incubator for 24 h in the same conditioning medium, followed by a 48-h incubation with the indicated concentrations of adropin. Cells were fixed with 4% paraformaldehyde in phosphate buffered saline. TUNEL staining was performed using an In Situ Apoptosis Detection Kit (Takara Bio, Otsu, Japan) as described previously [41,44,45,46,48]. Nuclei were co-stained with 6-diamidino-2-phenylindole (Dojindo, Kumamoto, Japan). The number of TUNEL-positive cells was counted in three fields of view chosen randomly from each sample.

### 4.7. Western Blotting

Aliquots of protein extracts (20 μg) derived from THP1 monocytes, their derived macrophages, HUVECs, and HASMCs were separated by 10% sodium dodecyl sulfate polyacrylamide gel electrophoresis, and then immunoblotted with specific antibodies raised against the following proteins: CD68, ACAT1, ICAM1, VCAM1 (Santa Cruz Biotechnology, Santa Cruz, CA, USA), ABCA1, collagen 1 (Novus Biologicals, Littleton, CO, USA), collagen 3, fibronectin, arginase 1, phospho (Ser529)-NFκB, α-tubulin, MMP2 (GeneTex, Irvine, CA, USA), MMP9 (EnoGene, Atlanta, GA, USA), elastin, MARCO, selectin E (Bioss, Woburn, MA, USA), PPARγ (Signalway Antibody, College Park, MD, USA), phospho (Thr202/Tyr204)-ERK1/2, phospho (Ser/Thr)-Akt (Cell Signaling Technology, Tokyo, Japan), c-Src (Bioworld Technology, St. Louis Park, MN, USA), PI3K, Bcl2, Bax, and β-actin (Sigma). The band intensity of the immunoblot was quantified by densitometry [39,40,41,44,45,46,47,48,49].

### 4.8. Animal Experiments

Animal experiments were carried out in accordance with the Guide for the Care and Use of Laboratory Animals published by the National Research Council, with protocols approved by the Institutional Animal Care and Use Committee of Tokyo University of Pharmacy and Life Sciences (No. L17-22, 24 April 2017). A total of 22 male spontaneously hyperlipidemic *Apoe*^−/−^ mice (BALB/c. KOR/StmSlc-*Apoe^shl^* mice) at 8 weeks old were purchased from Japan SLC (Hamamatsu, Japan) and fed a normal diet. At 13 weeks old, a high-cholesterol diet containing 1.25% cholesterol, 3.0% lard, and 1.625% glucose (F2HFD1; Oriental Yeast, Tokyo, Japan) was started [39,40,41,44,45,46,47,48,49]. At 17 weeks old, six mice were sacrificed as pre-infusion controls. The remaining 16 mice were divided into three groups of six, five, and five mice, and infused using osmotic minipumps (Alzet Model 1002; Durect, Cupertino, CA, USA) for 4 weeks with three doses of adropin (0, 5, 10 μg/kg/h), respectively. Doses of adropin were selected based on previous data [5,9,28,29]. Pumps were implanted subcutaneously into the dorsum, and replaced once every 2 weeks under medetomidine-midazolam-butorphanol anesthesia [45].

### 4.9. Animal Measurements

Four weeks after commencing infusion into *Apoe*^−/−^ mice, body weight and food intake were measured, and systolic and diastolic blood pressures were measured using the indirect tail-cuff method (Kent Scientific, Torrington, CT, USA). Blood samples were collected after a 4-h fast. Plasma levels of glucose, total cholesterol, triglyceride, and free fatty acid were measured by enzymatic methods (Denka Seiken, Tokyo, Japan) [39,40,41,44,45,46,48,49]. Plasma insulin level was measured by enzyme-linked immunosorbent assay (Ultra-sensitive mouse insulin ELISA kit, Morinaga, Yokohama, Japan) [45]. The HOMA-IR was calculated as fasting plasma insulin (pmol/L) × 0.139 (conversion to μU/mL) × fasting plasma glucose (mg/dL)/405 [45].

### 4.10. Assessment of Atherosclerotic Lesions

Before and 4 weeks after the start of infusion, *Apoe*^−/−^ mice were euthanized by exsanguination (total blood collection) under medetomidine-midazolam-butorphanol anesthesia [45]. The whole aorta was washed by perfusion with phosphate buffered saline and then fixed with 4% paraformaldehyde. The aorta was then excised from the aortic root to the abdominal area and the connective and adipose tissues were carefully removed [39,40,41,44,45,46,48,49]. The entire aorta lumen surface (the so-called aortic tree) and cross-sections of the aortic root were stained with Oil Red O for the evaluation of atherosclerotic lesions [39,40,41,44,45,46,48,49]. The immunohistochemical expression of monocytes/macrophages and VSMCs in atheromatous plaques were visualized by staining with antibodies raised against MOMA2 (Millipore, Billerica, MA, USA) and α-SMA (Sigma), respectively [39,40,41,44,45,46,48,49]. These areas of the aortic wall were traced by an investigator blind to the treatment and quantified by image analysis (Photoshop; Adobe, San Jose, CA, USA and ImageJ; NIH, Bethesda, MD, USA). In addition, the increased ratio of monocyte-macrophage contents (μm^2^)/VSMC contents (μm^2^) within the atheromatous plaques was regarded as a biomarker of plaque instability [41,45,46].

### 4.11. Statistical Analysis

Data are expressed as means ± SEM. Continuous variables were compared by unpaired Student’s *t* test for two groups, with one-way analysis of variance followed by Bonferroni’s post hoc test for ≥3 groups. Statistical analyses were performed using Statview-J 5.0 (SAS Institute, Cary, NC, USA). A value of *p* < 0.05 was considered statistically significant.

## Figures and Tables

**Table 1:** Characteristics and laboratory data of *Apoe*^−/−^ mice.

Parameter	17 Weeks Old	21 Weeks Old		
	Before	Adropin	Adropin	Adropin
		0 μg/kg/h	5 μg/kg/h	10 μg/kg/h
*N*	6	6	5	5
Body weight (g)	26.3 ± 0.7	28.5 ± 0.6 *	29.0 ± 0.6 *	28.7 ± 0.4 *
Food intake (g/day)	3.9 ± 0.5	4.1 ± 0.3	4.0 ± 0.1	4.2 ± 0.5
Systolic blood pressure (mm Hg)	92.3 ± 1.1	92.7 ± 1.7	92.2 ± 2.4	94.6 ± 1.8
Diastolic blood pressure (mm Hg)	70.4 ± 1.4	72.0 ± 2.0	69.9 ± 2.4	70.4 ± 0.5
Total cholesterol (mg/dL)	2136.7 ± 149.9	2224.0 ± 183.6	1845.3 ± 56.7	2170.0 ± 138.7
Triglyceride (mg/dL)	285.4 ± 52.7	295.1 ± 41.7	300.9 ± 46.0	308.3 ± 99.5
Free fatty acid (mEq/L)	4.2 ± 0.6	2.7 ± 0.9	2.6 ± 0.4	2.0 ± 0.8
Glucose (mg/dL)	258.6 ± 34.2	261.3 ± 33.9	248.8 ± 21.3	255.5 ± 24.1
Insulin (pmol/L)	30.1 ± 7.6	42.7 ± 20.8	76.4 ± 8.8	72.4 ± 45.7
HOMA-IR	2.8 ± 0.8	3.4 ± 1.4	6.6 ± 1.0	7.1 ± 5.0

Data are shown as the means ± SEM. Statistical analysis was performed using one-way analysis of variance followed by Bonferroni’s post hoc test. * *p* < 0.05 vs. before adropin infusion (17 weeks old).

## Abbreviations

ABCA1	ATP-binding cassette transporter A1
ACAT1	acyl-coenzyme A: cholesterol acyltransferase 1
AngII	angiotensin II
CAD	coronary artery disease
ECM	extracellular matrix
GPR19	G protein-coupled receptor 19
HAEC	human aortic endothelial cell
HASMC	human aortic smooth muscle cell
HUVEC	human umbilical vein endothelial cell
ICAM1	intercellular adhesion molecule 1
MMP	matrix metalloproteinase
NCEH	neutral cholesterol ester hydrolase
NFκB	nuclear factor κB
NOS	nitric oxide synthase
LDL	low-density lipoprotein
SRA	scavenger receptor class A
TNFα	tumor necrosis factor α
VCAM1	vascular cell adhesion molecule 1
VSMC	vascular smooth muscle cell
